# Strengthening field-based training in low and middle-income countries to build public health capacity: Lessons from Australia's Master of Applied Epidemiology program

**DOI:** 10.1186/1743-8462-6-5

**Published:** 2009-04-09

**Authors:** Mahomed S Patel, Christine B Phillips

**Affiliations:** 1 Master of Applied Epidemiology Program, National Centre for Epidemiology and Population Health, Australian National University, Canberra, ACT 0200, Australia; 2 Australian National University Medical School, Canberra, ACT 0200, Australia

## Abstract

**Background:**

The International Health Regulations (2005) and the emergence and global spread of infectious diseases have triggered a re-assessment of how rich countries should support capacity development for communicable disease control in low and medium income countries (LMIC). In LMIC, three types of public health training have been tried: the university-based model; streamed training for specialised workers; and field-based programs. The first has low rates of production and teaching may not always be based on the needs and priorities of the host country. The second model is efficient, but does not accord the workers sufficient status to enable them to impact on policy. The third has the most potential as a capacity development measure for LMIC, but in practice faces challenges which may limit its ability to promote capacity development.

**Discussion:**

We describe Australia's first Master of Applied Epidemiology (MAE) model (established in 1991), which uses field-based training to strengthen the control of communicable diseases. A central attribute of this model is the way it partners and complements health department initiatives to enhance workforce skills, health system performance and the evidence-base for policies, programs and practice.

**Summary:**

The MAE experience throws light on ways Australia could collaborate in regional capacity development initiatives. Key needs are a shared vision for a regional approach to integrate training with initiatives that strengthen service and research, and the pooling of human, financial and technical resources. We focus on communicable diseases, but our findings and recommendations are generalisable to other areas of public health.

## Background

Until recently, national capacity development for the control of infectious diseases in low and middle income countries (LMIC) was represented as an internal matter, in which richer countries acted as donors and technical experts – encouraging from the sidelines. The emergence and global spread of diseases such as SARS (Severe Acute Respiratory Syndrome) and avian influenza, and the threat of an influenza pandemic, have changed this paradigm. While disease control responsibilities are local, determinants of disease emergence and spread are often global, beyond the control of national governments [1-6]. Capacity development in LMIC – once a means to support poor countries to develop and manage their own disease surveillance and outbreak response systems [7,8] – has been re-conceptualised as a way of ensuring the integrity of communicable disease control in rich countries [9-11].

The International Health Regulations 2005 (IHR), endorsed by the 193 member states of the World Health Organization [12], reflect increasing global inter-connectedness. The regulations revise the 1969 IHR by broadening the range of, and creating a legal framework for, reportable 'public health events of international concern'. If poor member states are to implement the IHR effectively, many LMIC will require significant investments to improve surveillance and control of infectious diseases [9,12].

Low and middle income countries often have a porous patchwork of surveillance systems and inadequate resources to develop and implement effective early warning and response systems [9,13,14]. Deficiencies have been identified in four critical areas: health infrastructure; scientific methods and concepts of operation of surveillance and response programs; essential human, financial and technical resources; and international policies [9]. Many of these deficiencies are the result of long-term under-investment in health infrastructure [14,15]. Donor funding that focuses on targeted surveillance programs for specific diseases may de-stabilise the already fragile public health capacity of LMIC by, for example, drawing funds and professionals away from other essential public health activities [13,14]. For poor countries, the social and economic costs of existing diseases can be crippling. In sub-Saharan Africa, for example, infectious diseases account for 63% of all deaths, and 61% of DALYs (disability adjusted life years) [16]. Thus, while rich countries speak of the need to improve public health capacity in LMIC to ensure biosecurity, poor countries continue to speak of public health capacity as a way of addressing the dangers posed by existing disease within their countries.

Effective training programs are central to the improvement of public health systems and responsiveness. In-country field-based epidemiology training programs became a popular model of education through the 1990s and early part of this century [7,8,17-19]. Over 40 countries now have field-based training programs. These programs, while valuable, are often burdened with impossible expectations that they will by themselves "improve the health of a country's population by... providing essential public health services and ...strengthening the public health system's capacity and infrastructure" [20].

Counter to this view, we argue that effective capacity development must address some fundamental pre-requisites for strengthening broad-based public health in LMIC. Training should be integrated with the following parallel capacity development initiatives [21,22]:

• Strengthening organisational structures, policies and processes that guide and drive health services and essential research, including funding and national and international partnerships. Many LMIC have shaky knowledge-translation platforms [23]; there is often limited dialogue between academia, researchers, policy-makers and practitioners. Hierarchical health systems in which there are significant status differentials often mitigate against ready feedback of data and policy ideas from researchers and the field to policy-makers.

• Developing infrastructure and systems for service delivery across all levels of the health system, including systems for surveillance and response.

• Enabling adequate numbers and diversity of the health workforce across the health system. The workforce in many public health services in LMIC are depleted by internal and external migration, resulting in workers who are overworked and stressed, and often poorly motivated and supported within the system [15,24].

There are now Field Epidemiology Training Programs (FETPs) covering over 40 countries globally [8], most teaching applied epidemiology to control communicable diseases. Despite the global adoption of this educational model, little research exists into the ways in which training can become part of a larger capacity development endeavour. One of few such published studies was from Germany where the FETP was identified as one of six integrated strategies to strengthen disease control [19]. The other strategies were strengthening systems of surveillance, identifying short- and long-term research priorities, improving communications and interactions with program partners and constituents, and building international collaborations.

In this paper, we draw on the experience of the Master of Applied Epidemiology program based at the Australian National University to explore how a university-based training program partners and complements health department initiatives to improve public health capacity [25]. We argue that the tendency to reify "training" as distinct from other capacity development initiatives may be part of the reason FETPs in LMIC have difficulty bringing about transformative change in public health. We conclude by recommending inputs and pre-conditions necessary to ensure that an FETP can contribute effectively to public health capacity building.

## Discussion

### Capacity development

'Capacity development' can be defined as the deliberate effort to strengthen the ability of the health system (the health services, training and research sectors) to produce desirable outcomes [21,23]. Capacity development aims to deliver two outcomes: quality health services which respond effectively to changing community needs; and the conduct of essential research with appropriate uptake of research findings. The targets of capacity development are located at the individual, institutional, and sub-national/national and international levels [21].

### Public health training in low and middle income countries

Public health training in LMIC is provided through three different educational models – not all of them complementary. The university-based model of postgraduate training in public health draws upon the model disseminated by the Rockefeller Foundation of dedicated, prestigious training institutes, distinct from clinical training institutes [26]. There is a dearth of public health training programs in universities in LMIC. Australia, for example, has 18 Public Health Education and Research Program (PHERP) funded public health training programs through universities and institutions [27]; South Asia – with a population eighty times that of the Australia – has only 12 schools of public health [28]. In the African continent, covering a population fifty times that of Australia, there are only 50 schools of public health [28]. This model of education has been critiqued for providing classroom-based training of dubious relevance to 'real world' problems in LMIC [24,28]. The most devastating argument against this model of training, however, is the fact that there are too few institutions to produce sufficient public health personnel in LMIC. The continued dominance of this model as the most prestigious form of public health training in LMIC represents a diversion of resources from more effective forms of public health training.

The streamed training model has been used by UNICEF, WHO and other international aid agencies [24,29]. This model aims to train workers in specified areas of public health practice, such as tuberculosis or diarrhoeal and vaccine-preventable diseases. This model is usually taken up by junior health workers who are provided with a limited set of public health competencies. While it is cost effective in meeting its educational goals, the streamed training model creates a cadre of public health workers who are generally positioned too low in the health bureaucracy to improve health policy or essential research. However, this model can quickly, efficiently and cost effectively meet emergent issues at the grass roots – until higher level public health specialists come on site. This model is especially applicable in the more rural and remote areas of our region.

### Field-based training models

In field-based training models, the site of education is primarily the workplace, not the academy [7,8,17-19,25,30]. Drawing on vocational rather than professional education models, field-based training is responsive to the real-life needs of the workplace, encouraging flexibility among learners and the capacity to work as part of a public health team. Field-based training models are relatively new in LMIC. The field-based training model most used in LMIC for communicable disease control originated in the training programs of the Epidemic Intelligence Service, from the Centers for Disease Control (CDC) [7,8,17]. Field-based training was advanced in 1951 in response to the threat of biological warfare during the Korean conflict. The first CDC-based FETP in a developing country was set up in Thailand in 1980 [8,17]. The Rockefeller funded Public Health Schools Without Walls (PHSWOW) is a form of FETP offering an MPH qualification from a partner university [8,24,30]. It aims to produce generalist public health physicians rather than communicable disease specialists, and was launched in Zimbabwe in 1993, later spreading to other African countries and to Asia [8].

**Figure 1:**
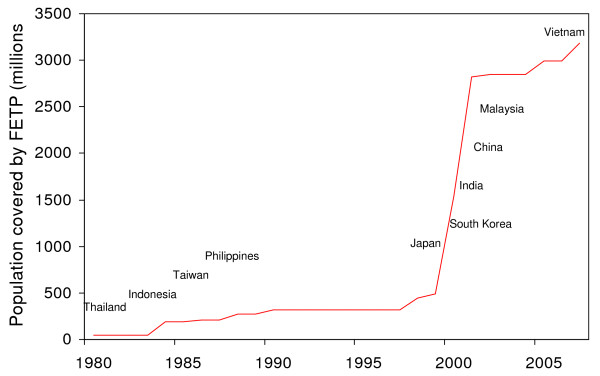
**Trends in Asian populations covered by an in-country Field Epidemiology Training Program 1980–2007**. Data sources [8,32].

Field-based training programs are the growth area in public health training for communicable diseases [8,25,31]. Figure 1 presents the trends in the size of the Asian population covered by an FETP from 1980 to 2007. Over 3 billion people in Asia live in areas potentially covered by an FETP student, compared to 45 million people in 1980. Although most FETPs in Asian countries restrict entrants to their own residents, Thailand's FETP has included students from other neighbouring Asian countries since 1997 [8].

According to member data of the Training Programs in Epidemiology and Public Health Interventions Network (TEPHINET), in June 2006, there were over 30 similar programs addressing the needs of almost 40 countries [32]. More than 85% of the programs initiated have been sustained for at least five years, and more than 75% of the graduates of programs in LMIC work for ministries of health after graduation [8].

### Australia's first Master of Applied Epidemiology Program

The Master of Applied Epidemiology Program at the National Centre for Epidemiology and Population Health, Australian National University (referred to as the 'MAE' in this paper) was the first such program to be introduced in Australia (in 1991). It offers two-year scholarships. Scholars are based typically in communicable disease directorates at the local, state-territory or national levels, but more recently, placement sites have included research institutes such as the National Centre for Immunisation Research in Sydney, the McFarlane Burnet Centre for Medical Research in Melbourne, the Queensland Institute of Medical Research in Brisbane and the Telethon Institute for Child Health Research in Perth. Two placements are reserved for Indigenous scholars each year; previous scholars have been based at Aboriginal Health Services (e.g. Winnunga Nimmityjah Aboriginal Health Service in Canberra, Inala Indigenous Health Service in Brisbane), or in the Office of Aboriginal Health in Perth. The Program is restricted to Australian residents, although four candidates (two from India, one each from Fiji and Timor Leste) were accepted through specific arrangements with their respective governments.

This MAE Program departs from its CDC parent program in that it is based in a university, and students continue to have both academic and field supervisors [25]. This is an unusual model among FETPs, which generally do not have academic support from a university, or a university qualification at the conclusion of training. The MAE has in some ways hybridised elements of Rockefeller's Public Health Schools Without Walls (PHSWOWs) and CDC's FETPs.

Training in the MAE is designed and implemented in the context of other capacity development activities across health services. The MAE Program gained much of its momentum in 1991 through the inauguration of the Communicable Diseases Network of Australia (CDNA) [33]. CDNA is a platform for national and state/territory directors of communicable disease centres, and selected infectious diseases experts, microbiologists and academics (including the MAE Program). Among the key goals on the network in 1991 were the revitalisation of systems of surveillance and outbreak response, and evidence-based policy development at state and national levels.

MAE students continue to participate in the fortnightly teleconferences of the CDNA, discussing emerging problems, outbreaks, and policy development. Senior directors of health institutions and MAE academic staff help identify student project priorities, collaborate in designing and conducting essential research, craft recommendations for action, and co-author publications. Examples of a selection of the co-authored publications mentioned elsewhere in this paper are cited [34-53]. The directors have a vested interest in identifying and implementing solutions. MAE students work across different levels within the health system, with research institutes, and across the non-health sector. Examples of studies conducted across sectors and published in peer reviewed journals with MAE scholars as first authors include studies with local/national food authorities [35,41], veterinarians and/or Department of Primary Industry [36,45,52], entomologists [40], local government authorities [38,48], Department of Immigration and Citizenship and the United Nations High Commission for Refugees [42,43].

These strategies help to strengthen the performance of the health system and health outcomes. At the same time, they support the design and conduct of research to generate evidence-based public health policies, programs and practice. This is a model of 'learning by collaborative problem solving' to address public health challenges perceived as being important by senior public health decision-makers and researchers.

Core competencies are based on the three core functions of public health: assessment; policy development; and assurance [54]:

• Assessment: aims to identify, define and prioritise public health problems and needs systematically, to respond to public health emergencies, to identify resources and to get commitment and inputs from people who will have to act on the results. It includes monitoring and analysing the health status of the population through the development, implementation and evaluation of surveillance and other health information systems.

• Policy development: aims to formulate and evaluate appropriate solutions including the development of effective policies, programs and public health practice. It includes synthesis and use of scientific knowledge and other factors prioritised by decision-makers and politicians for policy development.

• Assurance: aims to assure that policies, programs and plans are implemented. It includes appropriate systems of monitoring and evaluation to ensure effectiveness, efficiency, accessibility, and equity in population-based health services. It requires management and coordination of resources, building constituencies and identifying resources in the community.

Since 1991, the MAE Program has produced 139 graduates. As students, they have led or participated in over 200 outbreak responses locally, nationally or internationally, including Japanese encephalitis, SARS, and avian influenza in poultry and humans. In addition, each scholar analysed data from, and evaluated, a health information (usually surveillance) system; conducted at least one epidemiological study addressing a public health need; and presented at least one paper to a national or international scientific conference. Most graduates now work in public health positions in Australia, Asia and the Pacific. The learning and career trajectory of an MAE graduate is presented as an example in Appendix 1. In her projects, this student studied and developed systems to manage surges in public health needs; adopted local, national and statewide perspectives; undertook national and international networking; and integrated communicable diseases, primary health care, and environmental health.

The Master of Applied Epidemiology is an effective model for improving public health capacity in rich countries. The applicability of the MAE to LMICs, however, should not be assumed as an article of faith. In the following section we critically consider the MAE and other FETPs to identify aspects other than training that together drive capacity development.

### Beyond Field Epidemiology Training Programs, towards integrated capacity development initiatives

Field Epidemiology Training Programs have strengths in building workforce skills, particularly in comparison to the university-based model and the streamed training model. They can be customised by local health departments to local experiences, challenges and context to critically and creatively inform work. They enable very rapid response to disease threats – the effective coordinated response to SARS in south-east Asia in 2003 was in no small part due to the work of FETPs and graduates of FETPs working in the region [55]. Field Epidemiology Training Programs trainees have completed a wide range of studies with important outcomes; these have been documented elsewhere [7,8,17-19,56].

Nevertheless, they are not without limitations, and they do not always succeed in bringing about transformative change. There appears to be an ongoing need for these programs to have external support to undertake work at the level suitable for external scrutiny. To give one example, in 2005, MMWR produced a supplement containing 10 papers from a recent annual scientific conference of TEPHINET (the Global Network of Training Programs in Epidemiology and Public Health Intervention) [56]. Of the 10 published papers, nine were written by FETP graduates from LMIC, representing a professional constituency infrequently published in academic literature. Seven of the papers included an external consultant as co-author. The two that did not have an external consultant as co-author had local academic staff, from Uganda and Zimbabwe – both countries with the University-based PHSWOWs.

Finding a learning milieu that will enable FETP students to be integrated into capacity development initiatives can be challenging. In contrast to the conditions which obtain for the Master of Applied Epidemiology in Australia, field supervisors in LMIC are typically not as senior and often have direct operational responsibility for outbreaks and surveillance. Field Epidemiology Training Programs in LMIC are usually based in an operational unit in the health ministry, with little or no academic support [7,8,17,20]. Students therefore work in stressed health systems with insufficient personnel. Overwork and politico-cultural notions of "appropriate behaviour" at particular levels in a health system can limit the capacity of the worker to form the kinds of multi-level and cross-sectoral partnerships that support rapid responses or policy change. In our experience, the partnership of a sympathetic external support worker with recognised authority (in this case, the MAE academic supervisor – but the role could also be played by a supervisor from another valued institution in the country or the region) can help support partnerships and improved communication within the public health system. These, in turn, make more resilient health systems, able to respond flexibly to disease threats.

Curricula need to address "softer" management issues, in addition to biostatistics and communicable disease epidemiology [20,24,28]. It does not advance the health of a country for a student to produce an elegant case control study of the cause of an outbreak of diarrhoeal disease if there is no translation of this research into changed behaviours or environmental protection. These type of interventions are complex and costly, and require champion activists who will coordinate dialogue and actions across multiple government departments and the private sector, and often also with non-governmental organisations and international donor agencies. To know how to do this, the student needs a suite of skills that does not belong to the strict corpus of communicable disease epidemiology. If the student has been encouraged through their training to adopt an attitude of resignation to communicable disease outbreaks – documenting rather than responding – both the country and the donor agencies may well feel that their investment in FETPs has been wasted.

Finally, in relation to the International Health Regulations (2005) [12], FETPs must complement regional capacity development activities to help control trans-boundary spread of disease. In Australia, the MAE has been able to provide opportunities for students to observe and contribute to the global health protection initiatives of the IHR; but this is partly because of the seniority of their supervisors, and the willingness of the public health environment to respond rapidly to new and emerging communicable diseases. In LMIC, students may not be able to participate in these activities, either because the organisational culture precludes participation of 'students', or because the workplace is so preoccupied with immediate in-country needs that the larger international context of disease spread may seem secondary.

### Australia's role in capacity building in the Asia-Pacific Region

Australia has committed itself to supporting the improvement of communicable disease control in the Asia-Pacific Region. The 2006 White Paper on Australian aid highlights several trans-boundary threats to stability and development in the Asia-Pacific region, including emerging infectious diseases [57]. Since the first significant outbreak of avian influenza in 2003, Australia has committed more than $152 million through its aid program to boost detection and surveillance, emergency preparedness and disease control [58]. In its willingness to engage as a regional partner in the improvement of health systems, Australia is participating in what has been identified as a new approach to global health governance [3,59], in which international cooperation becomes a lever for improved health in the global community.

Communicable disease control is a flagpole endeavour for global health. Kaul and Faust cited communicable disease control as a global public good which simultaneously drives and instantiates international cooperation on health [2]. SARS, avian influenza, and the threat of pandemic influenza have all demonstrated the need for countries to work together. In this paper we have argued that FETPs in LMIC contribute to global health through improvement in their workforce skills, and by strengthening the performance of national health systems. In a globalised world, these contributions need to be scaled up.

Although the FETPs described in this paper have generally addressed communicable disease control, the principles are translatable to non-communicable diseases and environmental health. Studies beyond communicable diseases published in peer-reviewed journals by MAE scholars include Indigenous health [34,44,46,49,53], refugee health [42,43,51], mortality in internally displaced populations [50], maternal and child health [37,39,53], and diabetes [46]. Some of the points we have argued in support of the MAE model, particularly the engagement of interested and supportive academic supervisors, are used within the Public Health Schools Without Walls for generalist public health training. The internet offers a new platform for e-learning, mentoring and continued support both during and after the FETP [60].

The IHR underscore a need to strengthen FETPs to better contribute to global capacity. The MAE model has been an effective model in Australia to enhance public health capacity, and some of the reasons it succeeded may be of use in considering how to strengthen FETPs in LMIC. A key driver that will need to be considered in LMIC is the coordination of training activities with capacity development initiatives aimed at strengthening health and essential research systems. There is an increasing level of investment in the region from international donors, and technical and research agencies, particularly in response to avian influenza in birds and in humans, and the threat of pandemic influenza. The challenge for LMIC now is to navigate through the poorly coordinated capacity development activities across different government sectors and departments. These activities may duplicate or conflict with each other, and are often funded by different international agencies with their own vested interests and agendas. Ways in which Australia can support capacity development through and in parallel with training are presented in Appendix 2.

Education can be a driver for transformative change in health service function. To focus, however, on a training program as a public health capacity development measure in itself is to bury a good idea under the weight of expectation. The terrain is everything, even in public health capacity building. Supporting a milieu that will enable FETP students and the host country's health system to make the most of their training is the next challenge in capacity building in our region.

## Competing interests

The authors declare that they have no competing interests.

## Authors' contributions

MSP conceived the framework for this study and undertook primary data collection. MSP and CBP analysed and wrote the paper.

## Appendix 1: Case study of MAE scholarly activity by one scholar

Dr Catherine Bennet was a biological anthropologist, who undertook her two-year Master of Applied Epidemiology placement at the Hunter Public Health Unit in Newcastle, NSW. As an MAE scholar, she performed the following activities:

• Conducted a case control study related to Salmonellosis across three states, and identified imported fresh garlic and semi-dried tomatoes as the vehicle of transmission [47].

• Investigated an outbreak of gastroenteritis at a wedding in which 37/53 attendees were affected. Norwalk virus was implicated, and the mode of transmission was person-to-person spread during the function.

• At the invitation of the Commonwealth Department of Health and Ageing established a health surveillance system for 3397 Kosovars entering Australia with temporary safe haven visas in 1999. The system provided the infrastructure to link, analyse and disseminate health data at an operational level within health clinics, and for communications among the local, national and international collaborating agencies [42,43].

• Evaluated the NSW surveillance system for food-borne illness. Recommendations for a revised system were endorsed by the Directors of New South Wales Public Health Units.

• Conducted a systematic literature review on remediation programs for managing elevated blood lead levels in the vicinity of functioning smelters and other point sources. (Lead emissions from the lead and zinc smelter at North Lake Macquarie were an important challenge in the Hunter Region). The evidence supported a community-wide zonal approach to lead remediation. This report was used by the North Lake Macquarie Remediation Management Committee to inform expenditure of millions of dollars of the lead remediation funds.

• Assessed general practitioner (GP) reporting practices in relation to infectious diseases and met staff of all divisions of general practice in the Hunter Health Area to demystify the public health process. This led to the development of a resource kit for GPs to support more prompt reporting.

## Appendix 2: Ways in which Australia can support capacity development through, and in parallel with, training

### Training materials and support

Explore the use of new media, and in particular, distance learning materials to:

• Provide external, high-level support to students from experienced public health professionals and academics

• Develop, distribute and free-licence quality training modules

• Develop appropriate curricular materials on strategic management, approaches to change, advocacy and health policy formation.

### Preventing the brain drain

Support health workers from low and middle income countries to continue working in their countries by:

• Providing incentives through peer assistance and regular collaboration with the global public health community

• Partnering with countries in the region to underwrite the salaries of key workers in government public health units to counter economic incentives to work for NGOs.

### Using applied epidemiology to develop policy and practice

Support students and staff in public health units in low and middle income countries to be advocates for change by:

• Providing peer-to-peer support for workplace supervisors to foster the student's and supervisor's capacity to develop effective public health policy as a result of their investigations.

• Establishing regular meetings and e-contact between Field Epidemiology Training Program students to develop an internal peer network in which they will be encouraged to focus on outcomes of outbreak investigations or surveillance activities.

### Building collaborations

Support collaborative endeavours by:

• Establishing a number of clerkships for Field Epidemiology Training Program students or graduates in Australian institutions, and for Australian FETP students in regional ministries of health.

• Cooperative activities between countries within the region through developing peer-to-peer support from workers in other countries.

• Encouraging broader inter-sectoral communication; for example, with the animal health sector, by creating short-term clerkships in other health-related sectors outside the ministry of health.
